# A Comprehensive Framework for Human Resources for Health System Development in Fragile and Post-Conflict States

**DOI:** 10.1371/journal.pmed.1001146

**Published:** 2011-12-20

**Authors:** Noriko Fujita, Anthony B. Zwi, Mari Nagai, Hidechika Akashi

**Affiliations:** 1 National Center for Global Health and Medicine, Tokyo, Japan; 2 Global Health and Development, School of Social Science and International Studies, The University of New South Wales, Sydney, Australia

## Abstract

Noriko Fujita and colleagues offer a comprehensive framework for human resource system development, based upon experiences in three fragile and post-conflict health systems: Afghanistan, the Democratic Republic of Congo, and Cambodia.

**Summary Points**

- Responding to the global human resource crisis requires systems thinking if a more comprehensive approach to human resource management and development is to be achieved.
- We present a comprehensive and visible framework for human resource system development. This has been derived from the lessons learned in supporting human resource system development in three fragile and post-conflict health systems in Afghanistan, the Democratic Republic of Congo, and Cambodia.
- The efforts of development partners and the government typically concentrate on the “production” and training of health personnel, but this approach neglects other elements and often the necessary linkages between them. While there is potential value in focused forms of support, they will be much less effective, with negative effects on both systems and population health, when they are unbalanced, incomplete, or miss the necessary linkages between them.
- While the “house model” contains elements similar to the World Health Organization HRH Action Framework, some functions are extracted in order to draw more attention to them. Issues such as the legal and regulatory framework, coordination, and monitoring are often neglected. We also place particular emphasis on the linkages among elements by highlighting some core functions of human resource management (production-deployment-retention), or by separating the foundation components (policy and planning, finances, legal) as primarily the responsibility of the government.

## Background

Responding to the global crisis in human resources for health (HRH) requires systems thinking if a more comprehensive approach is to be promoted. This differs substantially from the traditional emphasis on pre-service education, in-service training, and personnel management [1]. The elements to be included in a more comprehensive assessment and response to HRH system development need to be derived from experience and evidence from the field, and should be validated into the future in different settings.

In post-conflict situations, large numbers of development partners, including United Nations (UN) agencies, international and local non-government organizations (NGOs), and various others, literally “rush in.” The situation is often characterized by a weak health system, and is complicated by the limited quantity and quality of human resources [2]. New governments and emergent ministries typically have limited capacity to manage all the tasks necessary for reconstruction. Development partners have their own mandates and agendas, and their support usually focuses on specific components of the HRH system even if they are operating within a functional aid coordination mechanism [2]. Disease-specific programs tend to receive priority, often promoting rapid expansion of service delivery through vertical programming. While human resources are recognized as a key, or indeed the key component of the health system, this vertical orientation to addressing needs may well weaken the overall functioning of the HRH system [3]. Innovative thinking that considers the wide range of actors and agencies, both internal and external, is therefore particularly necessary in post-conflict and fragile states.

The World Health Organization (WHO) and technical partners developed the HRH Action Framework in 2005. This Framework and set of tools are used to guide the situation analysis or planning process at the country level [4]. Field experiences in post-conflict and fragile states encourage us to ensure that key elements are identified so as to draw the attention of stakeholders to the prerequisites for effective reconstruction and the development of an HRH system.

## Objectives

The purpose of this paper is to present a comprehensive, engaging, and visible framework of HRH system development. This has been further developed from the lessons distilled from Japanese experiences of supporting HRH system development in three fragile and post-conflict health systems: Afghanistan, the Democratic Republic of Congo (DR Congo), and Cambodia.

Each country is briefly described in terms of broad context, health system challenges, human resource system developments, and emerging lessons. We also present a chronology and timeline that situates these experiences alongside one another (Table 1). Country experiences presented here are illustrative, not comprehensive.

**Table 1:** Chronology.

Afghanistan	
1979	Intervention by Soviet Union
1989	Withdrawal of Soviet Union following guerilla war
1996	Taliban seize control of Kabul
2001	Post 9/11: US-led invasion and fall of TalibanBonn Accord: Afghan groups agree to deal for interim governmentInternational community engagement with rebuilding the country
2002	MOH organizational chart revised at central and provincial levelsNational health resources assessment [5] revealed an extremely limited number of female health professionals (one female health worker per 50,000 general population)MOH priority: focus on midwives to overcome the high maternal mortality (1,900 per 100,000 live births) [7]
2003	Human Resource Task Force establishedProfessional categories delineated: testing, certification, and integration of health workers into the government systemMidwifery education curriculum developed: commenced in 16 midwifery schools
2004	General electionBasic Package of Health Services [12] contracted out for rapid expansion of health service deliveryHuman Resources formally established as a General Directorate in the MOHMidwifery education expanded countrywideMidwifery education: accreditation system for schools established [8]
2005	Local selection and contracting (to serve local community) of midwifery students commences in some provinces [9]
2008	Deployment of recent midwifery graduates: improved through local selection [9]

Democratic Republic of Congo	
1994	Rwandan refugees flee into DR Congo after Rwandan genocide
1997	Congolese rebellion ousts President Mobutu (in power since 1972)
1998	Instability caused by invasion of Rwanda and involvement of neighboring countries (Uganda, Zimbabwe, Angola, and Namibia)
2000	UN peacekeeping mission authorized (MONUC)
2002	Peace deal
2003	Formation of transitional government
2006	General election and new Constitution
2009	National health resource survey [14] reveals extensive need for health system reconstruction.National survey of training schools under the MOH and MOHE [15],[16] reveals uncontrolled overproduction of health professionals with minimal link to deployment.Annual health workforce report [19] shows the number of professionals reached the WHO regional standard (one nurse per 1,000 population)
2010	MOH develops National Health Development Plan [21] with the participation of international and national stakeholders
2011	National health workforce plan launched [22], focusing on strengthening human resource management

Cambodia	
1970s	Political instabilityKhmer Rouge regime (1975–1979): genocide and destruction of institutions
1980s	Vietnamese-led liberation: reconstruction of health systemHeightened emphasis on training of health workforce
1991	Paris Peace Accord and UN Transitional Authority in Cambodia (UNTAC)
1993	General election and new ConstitutionCountry rebuilding with international community supportInitial health workforce survey reveals overwhelming number of health workers, unregistered and without career structures
1995	Health Coverage Plan [25] sets the standard for staffing of facilities by professional categories
1996	Human resources development policy and health workforce plan [26]Educational system for nurses revised with a shift to quality education; midwifery education ceased until 2000
1999	Contracting in and out of health services to NGOs [25]
2000	Education of primary nurse/midwife re-started for rural health facilities, along with strategy of local recruitment and contracting of students, in order to meet standard staffing requirements in rural areas [26]
2002	Review of health service contracting health: support to MOH continued but contracting out ceased [33]
2003	Private teaching institutes commence educational programs for health workers
2005	Government budget with partner support for performance-based incentives [36]
2007	Accreditation system of training institutions put in place [29]High-level midwifery task force established in order to advance achievement of MDG4 [27]
2009	Minimum staffing achieved through the local recruitment and contracting of students: all health centers have at least one midwife [28]

Data sources for political chronologies: International Crisis Group [37],[38]; other references as per reference number.

## Process of Reconstruction in Three Post-Conflict Countries

### Afghanistan

Afghanistan underwent a prolonged civil war after the intervention of Soviet troops in 1979. The September 11, 2001 attacks, followed by the US-led intervention, resulted in the fall of the Taliban regime. The Bonn Accord formed the basis for an interim government (Table 1). In 2002, when the transitional government set about re-establishing systems and services in all sectors with the support of the international community, physical and social infrastructure was severely damaged, although little information was available about its extent.

One of the earliest activities in the health sector was the national health resources assessment. Undertaken in 2003, it mapped available resources (human, material, financial) in the country and surveyed teaching institutes to assess the educational environment [5]. These exercises revealed an extremely limited number of female health professionals (approximately one female doctor, one female nurse, and one midwife for every 50,000 individuals). This was a major constraint, as women in this Islamic post-Taliban society needed the permission of their families to attend health services; the absence of female health workers was a major barrier [6].

The extremely high maternal mortality ratio (MMR) (1,900 per 100,000 live births in 2000) prompted the Ministry of Health (MOH) to focus on midwives as a prioritized category of health professionals [7]. From 2003, through technical and financial support by development partners, midwifery education was promoted through the revised standard curriculum at 21 reopened or newly established schools [8]. Five schools were public and 16 contracted out to be run by international and national NGOs. To ensure quality, an accreditation system for the midwifery educational program was introduced at this time [8].

This expansion of training was, however, not linked to the subsequent deployment of midwives. The deployment rate of new graduates from one school in Kabul was less than 50% (AKDN program manager, personal communication). Awareness of these limitations led to new initiatives, including local student recruitment with an explicit agreement of post-graduation deployment to the health centers [9]. This local innovation was scaled up nationally and led to significantly higher employment levels and retention of trained midwives in rural communities [9].

According to the MOH and a United Nations Population Fund (UNFPA) situational analysis in 2008 [10], the number of personnel with midwifery skills (midwives and community midwives) had increased 3-fold from the 465 at the initial assessment in 2002. From 2003 to 2008, 991 (74%) of 1,337 newly graduated midwives had reportedly been deployed [10].

A Human Resource Task Force, chaired by the human resource department of the MOH, was established early in 2003 to facilitate coordination between development partners [11]. All development partners joined the task force, leading to harmonized activities that included the delineation of 17 categories of health professionals to be trained and recognized by the MOH and the introduction of a testing and certificate system to integrate health workers, trained on an ad hoc basis by NGOs during the conflict period, into the public sector [11].

In response to the need for the rapid expansion of health service delivery, the MOH began in 2004 to contract NGOs to deliver the Basic Package of Health Services [12]. Contracted NGOs recruited new staff outside of the government payroll and set up services rapidly, highlighting the need for a functioning human resource system [13].

At the provincial level, two factors constrained human resource management. The first related to MOH structure and capacity, both at the central and provincial level. The MOH department in charge of human resources concentrated on production, personnel management, planning, and information, while only a liaison officer was appointed at the provincial level, thus undermining engagement at that level [11]. Another factor was the limited capacity of the MOH due to slow progress in public sector reform. The Priority Reform and Restructuring (PRR) process was initiated in 2004 across the government to recruit competent personnel according to revised job descriptions and a new salary scale. It began from the mid-level managers at the central and provincial level, but progressed more slowly than anticipated, possibly reflecting political interference in the recruitment process (MOH staff, personal communication).

### Democratic Republic of Congo

DR Congo's conflict began with the Rwandan exodus after the genocide in 1994 (Table 1). After a peace deal in 2002 and the formation of a transitional government in 2003, the government has tried to stabilize this vast African country, but the people in the east of the country continue to live in fear.

Amongst the key health sector interventions were two surveys in 2009–2010, a national health resource survey and a survey of all public and private teaching institutes under the MOH and Ministry of Higher Education (MOHE). The national health survey [14] showed an extensive need for facility renovation; weak logistic, information, and financial management; and unequal distribution of doctors and nurses between urban and rural areas. Only 60% of 485 health zones had referral hospitals, with 60% of referral hospitals managed by the government and 33% by church organizations. The training school survey [15],[16] showed an increased number of low-quality private schools with associated overproduction of nurses and doctors, alongside an ongoing shortage of midwives and laboratory technicians, amongst others. The total number of teaching institutes under the two ministries had increased from 362 in 1998 to 551 in 2009. Only 14% of MOH teaching institutes and 48% of MOHE institutes met the accreditation criteria. Although professional categories, academic career paths, and accreditation standards for educational programs existed, both ministries were unable to implement the accreditation system during the conflict period.

The survey results above revealed the systematic problems of human resources in DR Congo. Training of health workers was not linked to deployment, one reason being uncontrolled production without workforce planning and monitoring [15]. There was a lack of information on available positions in both the public and private sectors. Only missionary institutions produce health professionals on the basis of their own service needs [15]. A third reason was that 8.9% of the MOH staff had already reached retirement age but continued in employment given the lack of a functioning pension system [17]. The World Bank is currently supporting Public Service Commission reforms in order to identify such staff and prepare processes to enable retirement [18].

The MOH's central level has three directorates in charge of human resources (human resources management, human resources production, and in-service training). Most provinces established a human resources department with similar functions. As a first step, these MOH and provincial department staff prepared and published, for the first time since the conflict, an annual report of the health workforce in 2009 with the support of technical partners [19]. This report showed that the number of health personnel per population (0.09 physicians and one nurse per 1,000 population), reached the WHO regional standard at least for nurses. In 2010, the health staff working at registered private clinics were also included in the annual report [20], which revealed that the vast majority of physicians (78%) and nurses (91%) are employed within the public sector. The extent of dual practice was not examined.

On the basis of these assessments and with the technical support of development partners, the MOH prepared the National Health Development Plan for 2011–2015 [21] and the National Plan for Human Resource Development for Health [22] as a sub-sector plan. Both plans were launched at the beginning of 2011. During the planning process, relevant ministries, professional councils and associations, labor unions, and development partners were involved. The MOH took this opportunity to establish a coordination mechanism among stakeholders with a focus on human resources as one of the priorities; however, little attention has been devoted to monitoring and evaluating the national plan [23].

### Cambodia

Cambodia has a unique history of genocide from the Khmer Rouge period (1975–1979). Health professionals were targeted and very few survived the Khmer Rouge. From the 1980s, the country started to reconstruct its health system with a focus on increasing the number of professionals [24]. However, it was only in the 1990s that Cambodia started developing institutional capacity, sustainable structures, and systems with the support of the international community [24] (Table 1).

The initial health workforce survey in 1993 by the MOH and WHO revealed 22,000 workers of varying abilities, almost all of whom were unregistered and without career structures [24]. There was a total of 59 categories of health workers trained, and the MOH tried to rationalize these into 29 equivalents [24]. In 1995, the MOH introduced health sector reform and launched a health coverage plan [25]. However, from the mid-1990s, development partner support for human resource development shifted its focus from quantity to quality [26]. From 1996 to 2003, the overall MOH staff numbers had dropped, decreasing by 10% for midwives and 5% for nurses, and staffing standards set for hospitals and health centers were not met, especially in rural areas [26].

Finding the balance between the quantity and quality of human resources required a system focus. To respond to the shortage of health workers in rural health centers, the MOH resumed the education of nurses and midwives working at health centers, with a strategy of local recruitment of students and a contract for post-training deployment [27]. Out of 936 health centers, the number of health centers without midwives decreased from 164 (18%) in 2006 to 0 in 2009 [28].

After 2000, political stability and economic growth brought about rapid growth of professional education in the private sector. The MOH and Ministry of Education, Youth and Sport (MOEYS), responsible for higher education in Cambodia, developed the policy foundation [29] and prepared accreditation standards for all health education programs. The accreditation system was enacted in 2007 [29]. This long, drawn-out process was an opportunity for the MOH and MOEYS to commence discussion and create a coordination mechanism to work together under the Council of Ministers [29].

Little attention, however, was devoted to the legal and regulatory framework for health personnel after the reintegration of the faction-affiliated health workers in the late 1990s [26]. Professional councils started to be established in 2000 (Medical Council) and subsequent years (e.g., Midwifery Council, 2006) for each category of professional [30]. While recent attention has focused on the licensing of health professionals, many related legal and regulatory issues are unresolved.

The financial aspects of human resources were substantial in the 1990s, and irregular pay and very low salaries forced staff to seek alternative sources of income for survival. Unofficial payments were common in the public sector [31]. As part of the health sector reforms, the MOH launched the Financing Charter of 1997, which promoted user fees along with an exemption system. User-fee income is managed by each health facility, and a portion is reserved as staff incentives and aims to stimulate performance improvements [32]. Government staff salaries increased annually by around 20% from 2003, but these salaries and incentives continued to be insufficient to support families, in view of Cambodia's high living costs [33]. Dual practice has been common for some time, and well-funded disease-specific programs through the UN and NGOs have attracted competent health staff out of the public sector, contributing to an internal “brain drain” [33].

Another element of health sector reform—contracting health services—was first trialed in Cambodia in 1999. The objective was to out-source the management of “operational districts.” This offered a model for other countries like Afghanistan to increase health service delivery output over a relatively short period, especially when applying performance-based payments as a stimulus for health service providers [13]. A concern of the transition from NGO-managed to government-managed health services relates to the challenge of ensuring acceptable remuneration for the staff of contracted health facilities [34]. The focus has shifted to performance-based incentives to retain staff [33]. Several schemes have been piloted since 2005 using government budgets and partner funds, but the appropriate policy, regulatory framework, and sustainable financial backup remain missing [35].

## Development of a Comprehensive Framework

Key lessons from the process of strengthening and re-establishing the health and human resources system were examined in three post-conflict countries (Afghanistan, DR Congo, and Cambodia) and are summarized in Box 1.

**Box 1. Lessons Learned from Analysis of Human Resource System Development in Three Post-Conflict Countries (Afghanistan, DR Congo, Cambodia)**

- Sound initial situation analysis is crucial to identifying the important contextual variables that influence human resource development: e.g., socio-cultural background, form and duration of conflict.
- Government and development partners typically concentrate on some components of the human resource system, often educational institutions and training, while neglecting others, such as the legal framework.
- Innovations that build on and support linkages across different components of the human resource system are more effective, as seen in recruitment and contracting of local students for deployment in Afghanistan and Cambodia.
- Balancing emphasis on quantity and quality of human resources is difficult without considering other contextual factors that affect the whole health system, such as the reforms to the health sector and education in Cambodia.
- MOH and related ministries typically have limited capacity while external agencies bring in significant resources along with their own agendas; coordination mechanisms that involve all players are key to reconstructing, developing, and monitoring the human resource system such as occurred with the Human Resource Task Force in Afghanistan and the engagement of national stakeholders in DR Congo.
- A meaningful, comprehensive, and visual framework that is easy to understand and identifies key components of the human resources system is of value.

In the aftermath of conflict and during the transitional and development stages, emerging governments typically require financial and technical support from development partners in order to strengthen the health system. As we have seen in all three countries, development partner support was typically concentrated on some elements of the human resource system but neglected others and their linkages. While these focused forms of support were of value, they were also somewhat unbalanced and incomplete, posing limitations on the ability of the health system to address population health needs [11].

**Figure 1:**
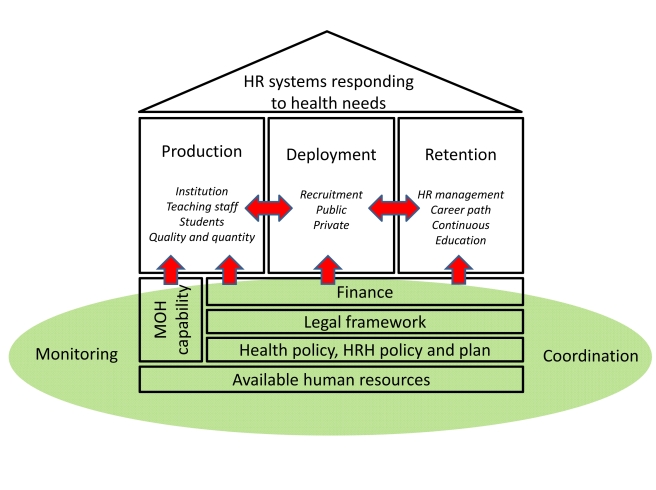
Human resources for health system development: analytical framework—the “house model”.

Based on the lessons learned from these three countries, we identified key elements of HRH system development and developed a framework, or “house model,” through which to promote its achievement (Figure 1). The value of the model lies in its being simple, visible, and easily understandable by all stakeholders.

While our house model contains elements similar to the WHO HRH Action Framework, some functions are extracted to draw more attention to them, including the legal and regulatory framework, coordination, and monitoring. We also put more emphasis on the linkages among elements by highlighting some core functions of human resource management (production-deployment-retention), or by separating the foundation parts (policy and planning, finance, legal) as primarily the responsibility of the government. Human resources are a key health system “building block”. Since the human resource system is a subsystem of the health system, elements identified are necessarily compatible with other elements of health system development.

The roof of the house represents the health system response (including human resources) to the health needs of the people, and all other components contribute to providing the human resource structures upon which that objective will need to rest.

Each country has different human resource challenges given their socio-political structures and experiences, health system structure, organization and resourcing, and the nature and duration of their conflicts. Interventions need to be guided by careful analysis and understanding of context. In all three countries, initial assessment (human, financial, material) was a first step with attention to available HRH as a starting point.

A house needs well-constructed foundations. In our model these include the national health policy and planning vision and framework, and the related human resources policies and health workforce plans, legal and regulatory frameworks, and finances. These elements are primarily the responsibility of central government, and involve several departments of the MOH plus other government ministries involved with planning, regulation, financing, and public sector reform. Since the MOH is the main producer, user, manager, and coordinator of HRH, and is also the avenue for advocating other civil service and administrative reforms required for effective functioning, MOH capacity is a core, and central, element of the foundation. In our experiences, the MOH and partners develop a policy and plan for HRH development, but there are a number of notable gaps. The legal and regulatory framework to implement the policy typically attracts little attention despite its importance in assuring the quality of health personnel. Other issues often neglected include oversight of the licensing or accreditation systems that link professional education to practice.

Production, deployment, and retention are the key elements of the processes of reconstruction, and correspond to the pillars of the house, standing on the foundations. As mentioned earlier, MOH and development partner attention is often directed to professional education, or “production” of health personnel. Where production is not linked to deployment, however, the mobilization of additional effort and resources does not bring the results envisaged. More importantly, developing links between production, deployment, and retention is key to system development and its efficiency, effectiveness, and equity.

Coordination, monitoring, and evaluation are shown as part of the base of the house in our model, as they cut across the entire HRH system. Gathering information about activities regarding areas of focus in terms of geography, topic, institution, and duration is an important step, as took place to some extent in both Cambodia and Afghanistan [2],[11]. Coordinating the shift from information sharing to joint decision-making and resource allocation helps shape and underpin the reconstruction and development of the HRH system [2].

HRH systems stand firmly within the social and cultural context of each country. Besides development partners, stakeholders are numerous, and include other relevant ministries, academic institutions, professional associations and councils, labor unions, and civil society organizations. In many post-conflict situations, a substantial reliance on external support is typical [36]: coordination, harmonization, and alignment of these resources is thus particularly important in relation to the HRH system. All stakeholders, however, need to be actively involved as the HRH system is developed and shaped, so as to make a sustained difference to health in the country in the future. Monitoring system development requires the identification of appropriate monitoring tools, including human resource information systems, which enable an ongoing assessment of the performance of different components of the system.

## Conclusion

We identified the core elements and their linkages for HRH system development, and represent this as the house model. By making the process dynamic, starting with the foundational blocks, the model highlights elements, thus simultaneously making them more visible to partners. The house model offers a visual symbol for reconstructing key elements within the health system—highlighting overarching functions required to address the needs of the community—and emphasizing how the underpinning components must fit together and reinforce one another if a sustainable structure is to be established.

The house model offers all stakeholders the possibility of facilitating collective work and agreeing upon the road map for assessment, analysis, and the generation of appropriate human resource policy and planning. We are continuing to work on in-depth case studies to identify constraints and lessons learned and to determine how best to support HRH system development in post-conflict and fragile countries. Using the house model may help guide HRH system development in some countries, and therefore the model becomes a valuable tool to help highlight the challenges, while imagining what and how things could be better.

## Abbreviations

DR Congo: Democratic Republic of Congo
HRH: human resources for health
MOH: Ministry of Health
NGO: non-government organization
WHO: World Health Organization
